# Care, career and health in times of digitalization—insights into the experiences of caring scientists

**DOI:** 10.3389/fsoc.2025.1647769

**Published:** 2025-10-28

**Authors:** Hanna Haag

**Affiliations:** Department of Social Work and Health, Frankfurt University of Applied Sciences, Frankfurt, Germany

**Keywords:** science, digitalization, care, health, COVID-19, home office

## Abstract

This article examines the experiences of caregiving scientists about the impact on well-being due to the increasing digitalization at work. The data is based on two qualitative research project using group discussions with scientists in positions of responsibility at German universities, which were evaluated using the documentary method and grounded theory. Contrary to the assumption that digitalization has a positive effect on health and well-being of those working in academia, the results show an increasing performance expectations in terms of permanent availability, which has a negative impact on the physical and mental health of those surveyed and exacerbates self-exploitation where physical boundaries are disregarded. The article contributes to the discourse on health and well-being in academia as well as on digitalization at the workplace with a special focus on care work.

## 1 Introduction

Well-being has not received sufficient attention in discussions about scientific work (Burian et al., 2022; Nicholls et al., 2022). This is surprising given findings showing that high demands and exhausting working conditions seriously impact the well-being of scientists, especially caregivers (Barkhuizen et al., 2014; Schnabel, 2006). The permanent availability of scientific manpower is required, which presupposes a healthy and efficient body. This exacerbates the already prevalent “care-phobia of science,” in which the ideal of the white, male and carefree “Homo Academicus” (Bourdieu, 1988) still dominates (Haag et al., 2024; Zimmermann, 2022). This worker is available 24/7, works 60 h per week, withstands enormous pressure to perform, and is globally mobile and flexible in terms of space and time. This ideal does not only involve a strong work ethic, but also a traditional image of masculinity and a certain image of elites. Following Joan Acker (1990, 2012) and her theory of gendered organizations we can argue that workplace structures, practices, and hierarchies are not gender-neutral but systematically reproduce inequalities, even in the academic environment. Within this framework, hegemonic masculinity (Connell, 1987) shapes organizational norms, privileging certain male identities who, due to their living conditions and physical constitution, can fit into neoliberal logics while marginalizing others who do not fit into these ideals. Those who advance submit to work and embody a “masculine habitus” (Bourdieu, 2002) in these “androcentric spaces.” This image of the ideal academic worker fits perfectly into an academic environment whose work processes are increasingly characterized by deregulation, competition, and output orientation at the structural level. These characteristics apply to many professional groups with hierarchical structures (e.g., politics, industry). Nevertheless the aim of this paper is to shed light on the situation in academia, as there has been a lack of research to date on the effects of increasing digitalization on the health of scientists with care responsibilities, especially in the early stages of their careers. The perspective on science thus serves as an example of professional spheres with similar structures in terms of work organization and career opportunities.

As other similar professional fields science is characterized by high performance standards, precarious employment conditions in the form of temporary employment opportunities. Many accept pushing themselves beyond their physical limits in order to remain in the system, as the rate of those who ultimately do not obtain one of the few permanent positions is high. The time pressure that young scientists below professorship level are under as a result of the Academic Fixed-Term Contract Act (WissZeitVG) is another influencing factor and, at the same time, a special feature of the German academic sector. The maximum limit of 12 years until completion of the postdoctoral phase creates particular pressure for early-career researchers, who must either find a permanent position in academia below the level of professorship during this period, which are extremely rare, or hope tob e appointed to a professorship within this time. Furthermore, many employment relationships are dependent on third-party funding and external donors. Young scientists often have to secure their own positions by writing project proposals, where they compete with others. Furthermore science continues to be strongly output-oriented, but the primary focus is on advancing one’s own career which is why there is fierce competition among researchers. Overall, scientists face high performance and evaluation pressure, particularly due to the high requirements associated with achieving qualification levels. Taking this in account it is not surprising that „in an environment where temporary, as-and-when contracts are more prevalent than permanent, tenured positions (…) individuals fear that by admitting to health conditions or disabilities they may be worsening their chances for employment” (Brown and Leigh, 2018, p. 987). Nevertheless, psychological stress and mental illness are very high among scientists of all status groups and, in some cases, higher than in other occupational groups. In addition to precarious employment conditions, the main factors include excessive workloads, increased productivity expectations, toxic dependency structures, lack of support, and poor work-life balance (Barry et al., 2018; Nicholls et al., 2022).

The COVID-19 pandemic further exacerbated the situation for many. Changes in working and care conditions, an increased risk of illness, as well as the loss of social networks have negatively impacted the well-being and health of many academics (Burian et al., 2022; Radtke and Burian, 2021). Fears about the future have increased, as have considerations of leaving academia (Berry et al., 2020; Haag and Gamper, 2022). Gender also plays a key role, as evidenced by the gender care and publication gap (Wegrzyn et al., 2021; Yildirim and Eslen-Ziya, 2021). Care work negatively impacted academic work and productivity during the pandemic (Haag and Gamper, 2025; Shalaby et al., 2021). If we want to address the care relationships of scientists, we must first define what care encompasses. Care is based on an understanding of humans as beings in need of care, which is not readily compatible with “modern categories such as autonomy, self-determination, and justice” (Schnabl, 2005, p. 57) and, in particular, with performance requirements such as those found in science, for example (Klinger, 2014). Understood as “social practice jointly shaped by the people involved” (Brückner, 2010, p. 50), care encompasses various forms. These include caring for children and other relatives, caring for colleagues, and self-care, especially in the context of health and personal well-being. Furthermore, the gendered and intersectional dimension of care work, which has already been highlighted in numerous feminist and intersectional discourses, must be taken into account (Gutierrez-Rodriguez, 2014; Hengelaar et al., 2021; Wichterich, 2023). The term “care” is used in a broad sense to describe the various forms of care that are important to the people involved. Care practices depend on various factors such as gender, age, and the background of the care providers and care recipients, and are therefore embedded in structures of social inequality. When considering care practices, these factors must be taken into account.

One key change in the wake of the COVID-19 pandemic is undoubtedly the increasing digitalization of scientific workflows (Getto and Zellweger, 2021; Rosak-Szyrocka et al., 2022) and the shift to home offices. In this paper “digital work” refers to working from home, as the intertwining with care work should be considered. The terms home office, flexible and digital work, or teleworking are therefore used synonymously. As society has already transitioned to post-pandemic normality, there is a greater need for research to analyze the relationship between teleworking and health (Castro-Trancon et al., 2024; Kniffin et al., 2021). Even before the pandemic, sociological research showed that digitalization can be seen as a cause of the intensification of work and flexibilization of working relationships (Carstensen, 2016; Väth, 2016; Hirsch-Kreinsen et al., 2015; Warmuth and Glockentöger, 2018). These effects have intensified during the pandemic. As Widar et al. (2022) determined, teleworking from home makes it more difficult to relax and increases irregular work hours as well as overtime work, which has a negative impact on well-being.

While the time spent on professions in essential areas such as publication, research, and third-party funding acquisition decreased, time spent on online teaching increased, which in turn caused the pressure of constant accessibility to increase. Telework has clear advantages, such as higher autonomy and flexibility, but can also affect one’s health and well-being (e.g., Buomprisco et al., 2021; Chirico et al., 2021; Beckel and Fisher, 2022; Crawford, 2022; Sevic et al., 2025; Felfe et al., 2022; Krick et al., 2024). According to ten Brummelhuis et al. (2021) the benefits of autonomy and productivity may be lost if employees feel pressure or are often interrupted by telework. While telework allows employees, especially those with young children, to improve their work-life balance, several factors can have negative impacts on health and well-being, such as stress-related issues due to the double burden of work and private obligations, lack of demarcation between work and private life, or blurred boundaries between work and leisure time (Niebuhr et al., 2022). It has become obvious that flexible digital work from home presents employees with the challenge of setting boundaries, otherwise work begins to permeate into one’s private space (Currie and Eveline, 2011; Ashforth et al., 2000). Currie and Eveline (2011) introduce the concept of extensification into the discussion, which means that “gainful employment extends into family and leisure spaces.” In this case, the “process of self-exploitation is often more evident than exploitation by the employer” (Currie and Eveline, 2011). Laß and Rüger (2024) show that the perception and experience of working from home during the pandemic depends on gender, commuting behavior, and, to some extent, the frequency of home office use. Castro-Trancon et al. (2024) also include a gender perspective in their analysis of the effects of teleworking on well-being. More than half of the studies they reviewed showed that teleworking has a negative effect on work-family interaction and work-family balance, and this effect is more pronounced for women. It becomes clear that although work-family balance is facilitated by increased flexibility, this situation results in pitfalls that can have negative impacts on well-being and health, especially in the case of childcare (Furuya et al., 2022). A recent review by Vacchiano et al. (2024) highlights the complexity of the interaction between telework and well-being and emphasizes the need for further research on how telework interacts with employees’ preferences, personalities, and life stages (van Dick et al., 2024).

Particularly in occupational groups in which a high degree of self-optimization, dissolution of boundaries, and personal responsibility can be observed, it harbors a risk of processes of instrumentalization and exploitation, as can be observed in modern society (Stadelbacher, 2010). They point to a type of work that, following Bröckling’s (2007) “entrepreneurial self,” can be described as “entreployee” (Pongratz and Voß, 2004), which is characterized by “self-control,” “self-commodification,” and “self-rationalization” (Pongratz and Voß, 2004). The individual, who is promised “autonomy, self-realization, and nonalienated labor” (Bröckling, 2007), “is increasingly confronted with the requirement of self-reflection to optimize their own occupational actions in a continuous adjustment process” (Warmuth and Glockentöger, 2018).

Furthermore, it is known that the ideal of the enterprising worker contains gendered and racialized assumptions, which in turn are based on life courses and introduce especially young women as the ideal neoliberal workforce due to their willingness to exploit themselves (Banday, 2025; Gill and Scharff, 2011; McRobbie, 2009; Scharff, 2016). Within this framework the individual carries the responsibility for failure of the self-optimization process. Working processes in the wake of processes like dissolution of boundaries, flexibilization, and digitalization are leading to a process of dissolution (Jurczyk, 2014; Kirschenbauer, 2015; Warmuth and Glockentöger, 2018). Heiden et al. (2021) state that although older studies argued that teleworking facilitates work-life balance (Fonner and Roloff, 2010; Percival et al., 2011), according to their results this does not seem to be the case for academics (Heiden et al., 2021).

As already discussed, there is an atmosphere of enormous pressure to perform, competition, and uncertain employment conditions in academia. Effectiveness and efficiency are becoming the central topos of scientific work in order to stay in the system and prove their perseverance. Digitalization processes can increase effectiveness through permanent availability and work independently of time and place, −at the price of breaking down boundaries and a high degree of self-discipline. Working from home is a suitable option. However, this comes at the cost of one’s own body and health. Existing studies show that the effects of teleworking are ambivalent. While some academics perceive home office and digital working as an experience characterized by stressors such as social isolation and a rapid shift to online teaching, others report that working from home offers more flexibility (Leal Filho et al., 2021; Watermeyer et al., 2021; Esteves et al., 2020; Widar et al., 2022). Due to the prevailing ideal of performance paired with the normal conditions of scientific work, the increasing shift of scientific activities to the home office or telework leads to the scientific subjects exceeding their physical limits due to the new technical possibilities. Taking Foucault’s theory of subjectivation into account, we can therefore speak of an instrumentalization of the body, which is used and employed (Foucault, 1977) in order to focus passionately and without exception on university work. Such a self-image is based on an understanding of autonomy, independence, impermeability, and constancy that perfectly fits into academic work (Zimmermann, 2022). In this arena of struggle, a form of self-discipline emerges that can be understood as a “conditioning, disciplining, and normalisation, above all towards oneself” (Maihofer, 2021). As we can observe in academia, working processes can be carried out in ways that are flexible and adoptable to private demands (Nickel, 2015).

At the same time, self-discipline also requires liberation from activities that restrict academic work, which include both self-care and care work (e.g., looking after children or relatives). The aspect of carelessness makes it clear that the conditioning of the body is closely linked to the notion of hegemonic masculinity (Connell, 1987; Maihofer, 1995); masculinity manifests itself in practices of subordination, complicity, and marginalization. Such a self-image is based on a heteronormative understanding of autonomy, independence, impermeability, and permanence, as Zimmermann (2022) notes. In short, it is about total dedication to science (Zimmermann and Weibel, 2020) as a site of performance where bodily sensations must be hidden or largely marginalized. The elimination of corporeality in its vulnerability (Butler, 2005)—not in its performance—is part of scientific self-discipline. At the same time—and this shows the ambivalence—science presupposes a certain form of corporeality as a resource and performer, which Campbell (2009) describes as “the corporal standard” and Zimmermann (2022) as “sportive competition.”

The male subject, who can—and must—submit to the “serious games of competition” (Bourdieu, 2002) seemingly independently and carefree, is contrasted with equally gendered care work, which is also addressed to the female gender in academia (Paulitz and Wagner, 2020). However, we showed in previous studies that fathers who actively participate in family life and see themselves as caring fathers are confronted with similar, if not the same, challenges as mothers and contribute to the move away from Homo Academics towards New Work (Haag and Gamper 2025). The question of health and well-being plays a key role in this context.

Nevertheless, there are few results on the effects of teleworking on health and well-being in academia (Heiden et al., 2021; Widar et al., 2022), especially with regard to care responsibilities we want to take into account.

## 2 Methods

The article examines the experiences of scientists with caregiving responsibilities in the context of increasing digitalization of work and focusses, how this affects their well-being and health in particular. The aim of the research is to investigate the effects of increasing digitalization on the health of scientists against the backdrop of academic careers and (often multiple) care responsibilities building on the experiences of the investigated subjects. Given that scientific work can be very flexible and location-independent (provided that physical presence is not required for laboratory work or similar activities), we can assume that the increased digitization of work processes in science has a positive effect on the health and well-being of scientists with care responsibilities, unlike scientists without care responsibilities, because due to their care work they have an increased need for flexibility, which digitization enables, for example, through the possibility of working from home. It can therefore be assumed that this particular group of scientists will find increased digitization beneficial and that the negative impact on their health will be minimized. A qualitative approach is particularly suited to exploring the experiences of scientists with caregiving responsibilities in the context of increasing digitalization of work, as it allows for a nuanced understanding of how these developments shape well-being and health. Qualitative research captures the subjective meanings, ambivalences, and coping strategies that emerge when professional and caregiving roles intersect with digital demands as well as with inequality factors such as gender or ability. It also enables the identification of situational dynamics—digital tools provide flexibility versus when they intensify stress- and uncovers aspects that may not be anticipated in advance, for instance feelings of guilt, shifting family negotiations, or new forms of awareness. For example, in the healthcare sector group discussions have highlighted how digitalization can simultaneously accelerate work and increase workload, often going unnoticed by management, thereby affecting employees’ well-being (Kaihlanen et al., 2023). In this way, qualitative inquiry offers a holistic perspective on the interplay of work, care, and digitalization, moving beyond isolated factors to reveal the lived realities behind statistical trends. The focus here is primarily on collectively shared experiences, which is why the group discussion method (Bohnsack, 2000) is particularly suitable, as it addresses collective orientations. Focus group discussions offer a particular advantage over quantitative methods in that they not only capture opinions, but also reveal how these opinions are formed, justified, and negotiated within a group. This allows us to see how these orientations relate to the experiences of the research subjects. While standardized surveys primarily measure distributions and frequencies, qualitative group discussions provide deeper insights into the subjective meanings, argumentative patterns, and social dynamics that underlie participants’ responses. As Morgan (1997) emphasizes, the strength of group discussions lies in the interaction between participants, which can generate data and insights that would not emerge in individual interviews. Furthermore, Kitzinger (1994) highlights that group discussions make it possible to explore unexpected themes and meanings that structured surveys might overlook. This makes them especially useful for exploratory research questions that aim to understand meaning and context rather than simply measure prevalence. Unlike quantitative surveys, this approach allows us to specifically map biographical experiences in order to find out how the respondents (in this case, early-career researchers with care responsibilities) (a) deal with the challenges of increasing digitalization in the context of care and health at the action level and (b) negotiate these challenges in intersubjective discourse. The connection to care work and well-being is an aspect that has not been taken into account in previous studies. At the same time, however, we know that the early-career phase leads to high health risks, which are exacerbated in the context of caregiving responsibilities.

In the following, I provide insights into two research projects that were conducted at the Frankfurt University of Applied Sciences. Since both studies focus on the connection between scientific careers and care work, data from both research projects is used. *Study I* “was founded by the Max-Traeger-Stiftung (February to July 2023) and investigated the effects of the pandemic on vulnerable groups at German universities (Haag et al., 2024). The study was carried out at German universities in six federal states. In eight group discussions (Bohnsack, 2000) 27 individuals (students and university stuff) were asked about their experiences during and after the pandemic. Their vulnerability was related to various factors: in addition to gender, the issues of caregiving, their state of health, and their origin played a significant role,

Allthough including, e.g., students with disabilities or care responsibilities as well as early career researchers with care work (fathers and mothers),. When recruiting the interviewees, we drew in part on our own networks and multipliers (e.g., disability officers, advice centers). The data was analyzed using content analysis (Mayring, 2007) and the grounded theory coding method (Glaser and Strauss, 1967) as well as the documentary method (Bohnsack, 2000). Coding was conducted according to grounded theory. Inductive codes were primarily assigned, focusing on the intersection of work, career, care, and health. Some passages were also analyzed in detail using the documentary method to identify implicit orientations, particularly those related to care and health, and thus also to identify underlying interpretive horizons such as “good fatherhood” or “good science.” In some cases, interview passages from different group discussions were interpreted simultaneously in order to identify overarching narratives.

*Study II* is conducted by the Hessian Ministry of Science and Art (October 2024 to May 2026) and investigates exclusively the experiences of fathers in science in East and West Germany. A total of five group discussions (Bohnsack, 2000) were conducted with early career researchers from the subject groups of social sciences and natural sciences/technical sciences The data was analyzed using the grounded theory coding method (Glaser and Strauss, 1967) as well as the documentary method (Bohnsack et al., 2013; Table 1).

**Table 1:** Two qualitative research projects as data basis.

	Topic	Duration	Location	Funded by
Project 1	Title 1	February 2023 until July 2023	Location 1	Max-Traeger-Stiftung (GEW)
Project 2	Title 2	October 2024 until May 2026	Location 2	Hessian Ministry of Science and Art (HMWK)

Source: own representation.

Even though the data material belongs to two studies and covers different survey periods, the data can be easily combined, particularly with regard to the topic of digitalization and health against the backdrop of care work during and aftermath of COVID-19 and also in terms of the methodological approach (both use Group Discussions). In particular, the situation of care work being performed using digital work processes in the home office and their effects on health and well-being forms an essential framework for the analysis. Health is not only understood as the state of health of the scientific subjects, but also refers to the health of third parties for whom scientists provide care. It is therefore about self-care and care for others.

For the presentation of results, we selected those participants in both studies aged 32–44 who, as early-career researchers, have care responsibilities (e.g., childcare, elderly care) for others or for themselves (e.g., due to chronic illness). Health is not only understood as the state of health of the researchers themselves, but is also analyzed in the context of caring for sick third parties. In study I selected two Discussions: one with three mothers (all postdocs at German universities, including two on fixed-term contracts) and the other with three fathers (all postdocs, two at German Universities and one at a non-university research institute). From Study II, we draw on two group discussions: Group Discussion 3 (four participants, two doctoral students, two postdocs, including one with a permanent contract) and Group Discussion 4 (five participants, three doctoral students, two postdocs, all on fixed-term contracts). However, not all discussion participants from the groups mentioned are named with references; rather, we have made a selection with a focus on health and well-being, which not all participants articulated equally. The selected individuals are not named with their real names, but with colors (e.g., Mrs. Red, Mr. Orange). Due to the fact that only one group discussion was conducted with mothers, and the vast majority with fathers, there is a gender imbalance in the sample. However, there is a gap in research regarding men’s perspectives on working from home, taking health aspects and concerns into account, which has so far been primarily studied for women (Mirchandani, 1999), which justifies this approach (Table 2).

**Table 2:** The persons mentioned in this paper.

Pseudonym	Care Responsibilities	Status	Employment	Project
Mr. Red	Two children	Doctoral candidate	University	Project II
Mr. Violette	Two children; chronical illness	Postdoc	Non-university research institute	Project I
Mr. Black	Two children, one with Down-syndrom	Postdoc	University	Project I
Mr. Blue	One child	Postdoc	Non-university research institute	Project II
Mr. Yellow	Two children	Postdoc	Non-university research institute	Project II
Mr. Orange	One child	Postdoc	University	Project II
Mr. Pink	One Child	Postdoc	Non-university research institute	Project II
Mr. White	two children	Doctoral candidate	University	Project II
Mr. Brown		Doctoral candidate	University	Project II
Mr. Green	two children	Postdoc, Lecturer with special responsibilities	University	Project II
Mr. Beige	Two children	Postdoc	University	Project II
Mr. Green	two children, caregiving for relatives	Lecturer with special responsibilities	Permanent	Project II
Mrs. Mint	One child; chronical illness	Postdoc	University	Project I
Mrs. Silver	Two children, care for relatives	Postdoc	Non-university research institute	Project I

Source: Own representation.

## 3 Results

The interviews in both studies were conducted in the post-pandemic period and thus show the effects of the COVID-19 pandemic on the perception of health and well-being in the context of scientific careers. One aspect that is already much discussed in the context of the pandemic and forms the focus of this article is the digitalization of scientific activities and the shift of these activities to the home office with simultaneous care work.

### 3.1 Experiences of paradoxical simultaneities in the pandemic

Although the interviews take a retrospective view of the COVID-19 pandemic, the interviewees remember the difficulties of the forced digitalization of work processes and their relocation to the home office with the additional burden of private care work. Many interviewees were faced with the challenge of continuing their normal working day while having to look after third parties (usually their own children). Work and care were thus directly intertwined during this time, as the quote from Mr. Red (GD3, Study II), father of two children and PhD candidate at a western German university at the time of the interview, shows. While Mr. Red’s wife, whose employer said “we have affixed a disinfectant dispenser to the wall here and we are putting on a mask,” was able and consequently had to continue her employment outside the home, he was at home with his children for a year and did “everything, i.e., school, kindergarten, cooking, cleaning, everything.” In addition to domestic care tasks, he had to continue routine work processes, such as teaching, which led, in his opinion, to great difficulties. He describes what he was told by the university:

*You’ve all got a laptop, you have got such a great headset, we are closing the place down, nobody can get in here, you do everything from home. The school and kindergarten did exactly the same thing and then I sat there. And then, I do not know, my daughter suddenly had some unstructured digital lessons in first grade, while I would actually have had a digital event. Now I cannot just put a six-year-old child in front of the computer and say, you are doing this while I’m doing my course. Apart from the fact that she would have needed my computer to do it, because at the age of six she does not have her own to do it, yes, and things like that (GD3, Study II).*

Mr. Red had no choice but to “survive,” which he describes as his “greatest achievement,” “but nothing came of it scientifically, nothing at all. I really spent a year, I did not read a single page, I did not write a single sentence, zero, because I could not.” It is clear from these statements that the use of digital technologies during the pandemic led to considerable multiple burdens and losses in terms of their own careers. Although it was apparently possible to reconcile work and care work, it led to increased stress and a lack of boundaries. Thus, Mr. Red was set far back in his academic progress with sole care responsibilities due to school and kindergarten closures while his wife worked. The fact that he had to deal with different digital processes—teaching and school—posed a particular challenge, so he switched to survival mode without having read a page or written a sentence.

One interviewee from the first study, Mr. Violette, father of two children, suffering chronical illness and postdoc at a non-university research institute, also reports on the dissolution of boundaries that he experienced during the pandemic due to the simultaneity of digital work and care work. Mr. Violette notes a general dissolution of boundaries between work and professional life in science because “you are somehow always sitting at the computer, you are actually always ready, so I always answer emails, then I watch soccer and then I sit down again, because there are three or four emails that still need to be written.” The pandemic further exacerbated this situation because “there was no I’m going home now, I was already there” (Study I, fathers). The lack of physical separation coupled with digital participation (here in the form of emails) blurs the boundaries between privacy and the work context. As it is always possible to use digital tools, and since these practices have intensified, it is much more difficult to draw the line.

In this section, what Becker-Schmidt, 1987 and Gudrun Axeli-Knapp (1990) have already identified as a double socialization of women’s simultaneity of reproductive and productive activity becomes particularly evident. Due to the pandemic, men, who had previously been able to devote themselves fully to their professional duties, were suddenly confronted with the reconciliation of care and work, leading to new negotiation conflicts for them, which, however, are already being intensively discussed in feminist discourses. Due to digitalization—in the case of the pandemic, the enforced home office—men no longer have the opportunity to escape domestic care work by working in offices, unlike before. Spatiality thus takes on a crucial dimension in the discourse surrounding the distribution of care work between the sexes. Who has the privilege of being able to leave the house to physically distance themselves from care work?

### 3.2 Flexibility and compatibility of scientific work processes in the aftermath of the pandemic

In addition to the experience of the COVID-19 pandemic, the interviewees in both studies focus more on the increase in digitalization of scientific work after the pandemic. They initially state that digitalization processes make it easier to reconcile work and care responsibilities by enabling more people to work from home and participate in digital work contexts. At this point, the discourse shifts from the enforced simultaneity to the enabled simultaneity of care and scientific work. The interviewees have learned new practices through the pandemic, which is particularly evident with regard to the male subjects. The question is whether this reflects a shift in priorities towards more care work in the sense of dirty work (Anderson, 2000) or whether the superficial participation in care work in the domestic sphere is limited to easily compatible activities, which ultimately demonstrate a prioritization of scientific work over care work. However, this cannot be investigated without a research design that focuses on doing care through an ethnographic approach. Instead, the focus is on the interviewees’ narratives and their self-presentation.

In the narratives, flexibility in science is considered with flexible working hours and flexible work locations. In contrast to other professional fields in which working with people (e.g., hospitals, service sector) requires a physical presence where employees must adhere to fixed working hours, the interviewees appreciate the freedom that working from anywhere and at any time gives them freedom. Especially for those who care for third parties, flexibility offers a high degree of compatibility with care responsibilities. For example, Mr. Black, father of two children, including a child with Down’s syndrome, and a postdoc at a University of Applied Sciences, stated:

*I work from home as often as possible and am only at the university when I’m teaching or when there are meetings that require my presence, because we have so much other stuff with our youngest that we have to organize. Not a lot now, but therapists twice a week plus visits to the doctor at least once a week or at least once a month. That’s three days that we have to divide up somehow. And then it’s kind of good that we know we are there and can manage it together, because we have another child who also wants to live his normal everyday life. And that has continued (Study I, fathers).*

Mr. Black differentiates between activities that he can do in his home office and activities that require his presence. While many science-related activities can be digitized, caring for children, especially the disabled child, requires a permanent presence. In the postscript, he indicates that he learned these practices during the pandemic and still continues them because it obviously enables him to achieve a good work-life balance.

Mr. Violette, who was working in a larger research network with different locations at the time of the interview, reported something similar. He identified a new routine that developed during the pandemic where “meetings are now all online. The only thing we still do is conferences. That means you travel around the country less, I have the feeling” (Study I, fathers). For him, as a father, it is now much easier to deal with the care responsibilities he has for his two children. Unlike the situation during the pandemic, the interviewees no longer see working from home as a way of breaking down boundaries, but as a space for freedom.

### 3.3 The flexibility trap of remote work and negotiating regulated working hours

Although the increasing digitalization of work processes during the pandemic seemed to relieve the respondents of work-life balance issues—they could work freely from home and commute less—the other side of the coin was also revealed in the interviews. For many, the increased flexibility also gave rise to a new problem: the question when work stops, and how they could avoid being permanently available. For example, Mr. Blue, Postdoc and father of one child, notes that he had to muster a high degree of self-discipline to tell his child while working from home: “No, watch out, I’m not going to put this off until after dark, I’m going to finish the application, I do not have time for you now” (GD4, Study II). Later on, the fathers in his group discussed the lack of distance between work and home, which directly resulted from the possibility of being allowed to work from home.

*Mr. Yellow: Yes, and you cannot externalize it, you cannot say I have to go to work, child, because otherwise my boss will fire me or my boss will fire me.*

*Mr. Blue: Yes, it’s really like that, because it’s just not like that.*

*Mr. Orange: Because it’s just not like that.*

*Mr. Blue: Yes, exactly. Yes, that’s what happens to me now. I have the luxury of being able to work from home a lot. That has a lot of light and shade. That’s exactly what happened when the little one did not feel like going to daycare, so she just stayed at home. But that’s the thing, you push emails into the evening or at night. And so, yes, you need a higher degree of… I do not know if discipline is the right word, but here’s a time slot, that’s where you start, that’s where you end. Yes, that’s true, it’s different when I have to go to the office in the morning because otherwise I’ll get a warning or something. That’s a different matter, yes.*

*Mr. Pink: Yes, it’s the same for me, because we are on the subject right now, we have the freedom, I’m also a work council member, there are also company agreements on how we can and may and should work remotely. And, no, of course we should not. And in principle, and from my boss’s point of view, they would let me work from home more than I do. But I just do not do that because the work goes better at the institute (GD4, Study II).*

Mr. Pink’s statement is particularly interesting. Due to his position as work council member, he could negotiate a different home office arrangement for himself. However, he prefers to work on site at the institute, which works better for him. Even if he does not elaborate, the discourse suggests that he is referring to the increasing blur of boundaries between work and private life in mobile work. This shows that office spaces function as an escape room from care work: Anyone who wants to work in a focused and efficient manner, which is assumed in science, must free themselves from care work. However, this presupposes that third parties bear primary care responsibility. The discourse being conducted here is thus a gendered discourse that suggests a kind of assistance in care work by fathers: They have the choice.

In group discussion 3 (Study II), the fathers also discuss the topic of working time arrangements and ask themselves what working hours are beneficial for them. As mobile work basically offers the opportunity to work at any time, they have to set limits for themselves, as the following interview excerpt illustrates:

*Mr. White: One of my supervisors in my dissertation, who also has a child and who set an example for me as his model, so to speak, said that at some point I noticed that when he writes me emails, he does so at half past one or two in the morning. I noticed that at some point. I asked him about it and then he said, yes, I just do it so that the afternoon and evening somehow belong to the family and then when the child is in bed and so on and then I sit down again and do something. I’ve tried all sorts of things. I once got another tip: get up at four in the morning and then do something somehow, and then I realized that getting up at four in the morning wasn’t really my thing. And then I thought I’d give it a try and that was more like it.*

*Mr. Green: But you have to get up in the morning, do not you?*

*Mr. White: Yes, but it works. It works for a while.*

*Mr. Green: Wow, I do not think so.*

*Mr. White: So… [laughs].*

*Mr. Green: I think it’s really crass. Because I would say that has changed too. I no longer work nights. Because I know I have to get up at half past six the next morning. No, I think it’s crazy that you can do that. I’d be sick all the time, I think.*

*Mr. White: No, that’s fine, at least for a few days. Then I need another two nights that I can sleep through to catch up a bit, and of course I cannot do that until the end of the semester. And then you have to differentiate a bit between the semester and the lecture-free period.*

*Mr. Beige: I have very clear boundaries, I think. I’m actually more of a nine-to-five worker and would like to only be active with work topics during this time and then stay away from them. I’m also a trade unionist. So I wish it would work like that, yes, and then I always have to realize, well, it does not really work out that way. But it’s exactly this working in the evenings, even working at home. I think it’s always such an imposition. I’d always rather have everything separate. Of course, sometimes I do not do it that way, but ideally I always try to keep it at bay so that I can do it at times that are really planned, which for me are also legitimate times, like during the day, or plan it differently, because then I tend to get sick more quickly. So I can do it for a short time and then I always get the receipt. So yes, I respect that, but at the same time I know it would not be feasible for me (GD3, Study II).*

Mr. Green and Mr. White disagree about working at night. While Mr. White sees this as an option for combining care work and academic work, Mr. Green considers it as a health risk. It would make him ill. Although he has apparently also worked at night at times, he no longer does so. However, Mr. Green is the only person in the group who has a permanent position as a lecturer at a university. At another point in the interview, he introduces the change from a part-time to a permanent position as an opportunity to organize his work according to his needs. Mr. Beige positions himself between Mr. Green and Mr. White. He also sets clear boundaries for himself. As a trade unionist, he pursues the ideal of doing science as a nine-to-five job and largely “keeping the stress at bay” by working regular hours. The reason he gives is that otherwise he would quickly fall ill. Working without boundaries is therefore related to one’s own health. On the one hand, Mr. Beige expresses respect for Mr. White, in which the implicit orientation towards the performance standard becomes clear, but on the other hand he knows that this “would not be feasible for him.” This shows the ambivalence in dealing with the performance standard: all three have to position themselves in relation to it and cannot avoid thinking about it, regardless of how they do so.

### 3.4 Going beyond the limit—the effects of telework on health

As was made clear in the last quote, digital work is directly related to health issues and is negotiated ambivalently. The other data in the sample also repeatedly reveals tensions regarding the handling of health-related aspects in the context of telework. A central feature of the debate is that, on the one hand, the interviewees see the possibility of participating in work life even in the event of illness, an advantage for them in terms of compatibility. On the other hand—and this aspect is particularly important in the context of the perspective raised—they state that this opportunity encourages them to ignore signs of exhaustion and health problems, which would not be possible in an analogous situation. The interviewees engage in a form of self-criticism, as this quote shows.

*For example, I had covid and wasn’t fit for a long time, so I asked myself whether I should go into the city or work online. The idea that I could skip it did not occur to me at all. And I find that crass. You just do not drive there, you do not drag yourself to the office, you just drag yourself to your desk (Study I, mothers).*

Mrs. Mint, a substitute professor with a long commute (over 500 km) and mother of a small child, expresses her inner conflict. She suffers from migraines and a mental illness. She manages to minimize her commute by working from home but accepts that she has to drag herself “sick” to her desk in order to participate digitally in work contexts (especially teaching, as she has a very high teaching load as a substitute at a University of Applied Sciences).

However, this leads to the astonishment of a colleague who says to her: “Watch out, you are ill, why do not you give it a miss?” (Study I, mothers). With regard to her chronic illness, Mrs. Mint states that she cannot spend so much time on the PC because “a lot of screen time is a trigger for my migraine.” The apparent advantages of digital participation in turn lead to health impairments, so as Ms. Mint concludes: “Well, I think that despite all the advantages, I also have the feeling that it actually puts additional pressure everywhere, yes, because you can actually be there” (Study I, mothers).

Mrs. Silver, who has a permanent position at a research institute, is the mother of two children and is in a dual-career relationship (her husband has a professorship in city X, they both live in city Y and she herself commutes to city Z, which is a little further away), describes similar experiences. For Mrs. Silver, working from home is initially a relief in the context of her care work. In her case, it is not only about caring for her children, but also about caring for her seriously ill father. During the conversation, she has an internal conflict over the question of how much work is reasonable and feasible in the following situation:

*So I worked from home with my dying father. I sat at my laptop and my father slowly died next to me. And it just would not have been possible to cover all this time on vacation because, I do not know, I would have had to take a lot more vacation than I would have had. That was a total concession. But it was always mentally challenging for me to see if it was somehow feasible to continue working here when these were probably the last hours with my father. I found that morally it was the best option, but it still wasn’t a good option (Study I, mothers).*

The deathbed becomes a place where care work and productive work can be combined—but at the expense of one’s own mental health. Here too, even if not openly expressed, the norm of performance pressure is something the interviewee cannot escape, even in an extreme situation such as caring for the dying—at the expense of her own body and health.

It is striking that the respondents repeatedly weigh different states of health. In the mothers’ discussion, a “traffic light system” emerges, which the interviewees follow in their everyday lives:

*Mrs. Mint: And I notice that I have escalation levels like this. Presence is actually the desirable maximum that works when the child is healthy and everything is going well. Then there’s one below that, online teaching, that’s not necessarily… I cannot do it all the time, but it’s okay. And then a bout of illness is okay or the child is there a bit. And then there’s another bout of sickness, everything implodes, and nothing works anymore. And I have the feeling that this is how we organize our everyday life, always in this total escalation stage.*

*Mrs. Silver: It’s like a traffic light system or something.*

*Mrs. Mint: Exactly, yes. And I find that violent. So that wasn’t at all clear to me. But now there’s just one more thing between presence and sickness, and that’s digital, at least for me, in my head (Study I, mothers).*

Whereas previously there was a trade-off between being healthy (attendance is possible) and being ill (attendance not possible), online teaching offers a third option, which places the decision even more in the hands of those affected. Unlike face-to-face teaching, it is also possible to complete the course if you are ill or have a sick child. In the case of online teaching, “a little sick is okay,” both in relation to oneself and to the child, which is misleading, because a child is either there or absent. The decisive factor is that, depending on the state of health, a decision is made as to whether teaching activities can take place in person, online, or not at all, which is only the case when the traffic light is set to “completely ill” and therefore, red. For the respondents, this results in a decision-making dilemma that they are repeatedly confronted with. While there appears to be a clear standard for in-person teaching that allows them to suspend teaching in the event of an illness, there is no such rule for online teaching. In this case, the sense of responsibility coupled with the possibility of online participation implies that the interviewees do not necessarily consider being ill as a reason to suspend their teaching activities.

In their discussions, the fathers also repeatedly weigh different states of health against each other and differentiate between their own health and the health of others, as the following excerpt from group discussion 4 by Mr. Blue shows:

*Yes, your own health is one thing. If you fall ill and something important comes up, you fight your way through it. If you are not completely exhausted, if you do not have to go to hospital, you fight through it. But when the children get sick, it’s no longer in your hands. In that case, you have to get your partner to agree to do it, because the children can no longer go to the institution and that’s a good thing. Then you have to decide who will stay at home. And that can be a challenge. If both partners have important appointments, the famous negotiations start. At least for us. How best to organize it. We try to organize it. I also have the option of working from home here. It often comes down to that. My colleagues understand that I work from home. Then the working day stretches from six in the morning to eight in the evening, with long breaks in between. That’s what the working day looks like (GD4, Study II).*

This statement makes the distinction between one’s own body and the bodies of others particularly clear: while one’s own body can be controlled to a certain extent and one can ignore its signs up to a certain limit—in this case the stay in hospital when one is “completely finished”—and fight it, the body of one’s own children represents an obstacle to continue working, since someone must take care of them. This is based on different notions of vulnerability the interviewees show: one’s own body is considered less vulnerable because one can exercise control over it and dispose of it, whereas the bodies of others are more vulnerable and less controllable. The way out of this dilemma seems to be working from home so that you can be with your sick child as well as at work—with an extended working day as a consequence. The destructive attitude towards one’s own health is questioned surprisingly little; here the individual seems to be responsible for himself. Caring for others is still seen as the norm to be followed.

Mr. Yellow in group discussion 3 also says that he cannot do anything until he is really ill, which makes it easier for him to weigh his options. “Intermediate stages are more difficult. Tiredness or being slightly ill.” The interviewee admits that he has not yet found a good way to deal with this, but usually tries to get something done anyway. “It often turns out to be nothing or not that great” (GD 3, Study II).

Mr. Green is also familiar with these negotiations, although he has largely distanced himself from the performance standard by being made redundant. However, he takes his teaching duties very seriously, which is why he sometimes continues to work despite being ill. In this case, he enjoys the advantages of digital teaching, because “that’s just the joke, it works. I can email the students 2 h in advance, we do it digitally. They join in” (GD 3, Study II). Mr. Green draws a comparison with other professional fields and notes that although there are some office jobs where this arrangement is possible, in a similar way to knowledge custody, there are also many professional fields “where it would not work. I would have to be on site and do something. However, it is precisely this possibility that leads people to ignore illnesses, at least up to a certain level” (GD 3, Study II).

The dilemma in which the interviewees find themselves becomes clear from their statements: Digital participation options bring opportunities, but at the same time entail the risk of overstepping boundaries and opting for digital working at the expense of one’s own health. None of the interviewees questioned the individualization of responsibility at this point, and no structural solutions to the problem were sought. The health issue appears to be an individual task that everyone must solve for themselves.

The interviewees can only free themselves from this situation when their own body’s vulnerability can no longer be ignored, when their own health is at stake, which prompts them to draw clear boundaries. For example, due to his chronic illness, Mr. Blau “sometimes cannot avoid” simply “taking it seriously and accepting it as a boundary” (GD 3, Study II). For him, this indicates a learning process that extends to the professional group of scientists, which he attributes to the fact that there is “always this tendency to get out of hand.” In the postscript, it becomes clear that he is primarily referring to structural aspects: “You are always being asked to do things and the employment contracts are not entirely clear. Where do the activities end, where do others begin?” (GD 3, Study II). What the interviewee is referring to is the dissolution of boundaries in work under precarious working conditions, a double pitfall that requires the scientific subject to draw clear boundaries. Once again, as a scientist himself, he is required to negotiate and adhere to these boundaries in the sense of a learning process, although he highlights systemic errors.

A similar statement is made by Mr. Brownin group discussion 1, who clearly distances himself from multiple burdens because he is of the opinion that he “cannot function twice.” He refers to “not being able to be on parental leave and sit at the computer at the same time,” thus demanding clear boundaries for himself, which he also places on the individual responsibility in the sense of a decision. He comes to the following conclusion:

*You’re also blaming yourself for this. (.) If I’m with the child now, then I cannot do anything else. That’s not for me, I do not want to burden myself with it psychologically. I just do not do it then. And if my life turns out differently, in the long term, then it will turn out differently (GD 1, Study II).*

For Mr. Brown, it is clear that his own health comes first and he does not accept the double burden that others in the group accept because he perceives an impact on his mental health. In the last sentence, he makes his position clear: he is not prepared to take on everything for the sake of his academic career and, if necessary, accepts having to take a different path. However, criticism of the structures of the scientific system is only implicit. It remains a decision for the individual to submit to these structures or to resist them — with all the consequences.

## 4 Discussion

As we can see from the data, care work, digitalization, and academic performance are closely linked. In terms of home office interference during the pandemic, many interviewees recognize a lack of appropriate work space, lack of distance between work and personal space, having children at home or other care duties, and blurred boundaries between work and private life.

Without structured working hours and breaks, and due to pressure, such as deadlines or high workloads in research projects, permanent digital accessibility and constant access to work in teleworking is often perceived as boundless and requires individual strategies, self-discipline, and mental flexibility (Widar et al., 2022), as the interviewees explain. Even though it was assumed that these aspects would take a back seat to the flexibility gained, precisely because the study group has an increased need for flexibility due to their caregiving activities, they are no less challenged to set boundaries. Flexibility also leads to self-exploitation among this study group. With increasing digitalization, it becomes even more necessary to set boundaries in the interests of all work subjects and in the interest of the subjects scientists care for. Their physical and mental health plays a role in this setting as well. If some people signal that they are available in the evenings or on vacation and that they are willing to ignore their own boundaries to be productive, there is a risk that this will gradually become a standard (Wagner, 2013), also among care givers. And at the same time this interplays with their care duties.

Above all, the data shows the ambivalence between the positive effects of digitalization and the challenge of not using it to the detriment of one’s own health, especially in the context of balancing career and care demands. Although increasing digitalization seems to make it easier to reconcile science with caring activities and provides more flexibility, new risks and challenges need to be examined more closely. Challenges include increased demands on drawing boundaries, withdrawing from permanent availability, and taking care of themselves and others. Lastly, the body’s vulnerability also plays an important role in this context. Due to the high demands and pressure to perform, a healthy, efficient body is a prerequisite in the scientific field. The self-exploitation that many scientists experience pushes them beyond the limits of their bodies and their resilience. This can result in health risks such as burnout or other chronic health-related issues. In the context of physicality and health, a distinction is made between one’s own body and that of others. It is evident that the people we interviewed apply different standards to themselves and are more willing to go beyond their limits, ignore physical signals, and overlook factors that cause illness. Scientists thus seem to consider themselves as disembodied subjects of work, functioning in the role of performer. It is particularly evident that one’s own body is made aware of in the context of its own vulnerability (Butler, 2001); only when its integrity is threatened does it become an object of concern. It therefore requires the bodily experience of being a body (Plessner, 1981) in the form of its vulnerability—such as a depressive episode, a migraine attack, or a dying father—in order to physically feel, and at least partially interrupt, the heteronormative performance ethos.

Even though the pandemic has changed the situation of men with care responsibilities, and digital technologies are influencing the ability to combine work and care work, the data clearly shows that this continues to be a matter of combining care with academic work, and not the other way around. Academic work continues to have priority and is not suspended, as is necessary for numerous part-time women who are primarily responsible for care. Rather, it is a kind of add-on in the sense of multitasking: they can manage it, as long as they set clear boundaries. None of the male interviewees explicitly spoke of a dilemma of having too little time to care for their children due to the many responsibilities at work that now also arise at home. In contrast, the mothers present the traffic light system and (have to) prioritize care work over productive work when the light is red. In the interviews with male respondents, health impairments caused by digital work are presented as a result of the simultaneity of care work and paid work. However, this is not about the blurring of work boundaries, but rather about care work being added as a new challenge, whereas for mothers, paid work intrudes into the sphere of care work.

## 5 Conclusion

The article explored how scientists with care responsibilities cope with increasing digitalization (especially working from home) and how this affects their health. Empirical material from two qualitative research projects on academics at German universities was used to answer this question. Previous research on the health risks of increased digitization processes among early-career scientists has not taken the aspect of care work into account. As a result, no findings have yet been obtained on the connection between increasing digitization in science, scientific careers, care activities, and health. This study explored this connection using a qualitative interview study. In this way, the findings contribute to the debate on care, physicality, and vulnerability as forms of social inequality in academia and sharpen the debate on the extent to which digitization processes really increase flexibility or rather contribute to further blurring boundaries, especially from the perspective of care work.

Due to the increased need for flexibility in terms of balancing care responsibilities and work life, we initially assumed that digitalization would have an exclusively positive effect on their health, because digitalization (e.g., through flexible working hours, working from home, less travel) creates the best possible structures for work-life balance. Contrary to this assumption, the results of the study show that even among caring scientists, especially those in the early stages of their careers and with intersecting vulnerabilities (e.g., childcare, ability, gender), the increased digitization of work processes is experienced as quite ambivalent. This, in turn, has an impact on their well-being and their mental and physical health, especially for early-career researchers, who are under particular pressure due to the high demands of scientific careers and the fact that their continued employment depends on their output. It was shown that several people surveyed see themselves at risk of health problems due to various factors that intersect with each other. For example, the high-performance expectations in the context of scientific career planning, life circumstances such as parenthood, supporting relatives in need of care, commuting times, or pre-existing chronic health conditions influence the interplay between scientific careers and personal health in case of care responsibilities. Above all, the data shows the ambivalence between the positive effects of digitalization and the challenge of not using it to the detriment of one’s own health, especially in the context of balancing career and care demands. Although increasing digitalization seems to make it easier to reconcile science with caring activities and provides more flexibility, new risks and challenges need to be examined more closely. Challenges include increased demands on drawing boundaries, withdrawing from permanent availability, and taking care of themselves and others. Lastly, the body’s vulnerability also plays an important role in this context. Due to the high demands and pressure to perform, a healthy, efficient body is a prerequisite in the scientific field. The self-exploitation that many scientists experience pushes them beyond the limits of their bodies and their resilience. This can result in health risks such as burnout or other chronic health-related issues. In the context of physicality and health, a distinction is made between one’s own body and that of others. It is evident that the people we interviewed apply different standards to themselves and are more willing to go beyond their limits, ignore physical signals, and overlook factors that cause illness. Scientists thus seem to consider themselves as disembodied subjects of work, functioning in the role of performer. It is particularly evident that one’s own body is made aware of in the context of its own vulnerability (Butler, 2001); only when its integrity is threatened does it become an object of concern. It therefore requires the bodily experience of being a body (Plessner, 1981) in the form of its vulnerability—such as a depressive episode, a migraine attack, or a dying father—in order to physically feel, and at least partially interrupt, the heteronormative performance ethos.

A debate from a critical perspective (e.g., disability studies, fat studies, critical whiteness studies, gender studies, postcolonial studies) on who and what a “healthy body” actually is (or is not) regarding personal health would be important here. These perspectives would ask intersectional questions about power and domination-shaped inclusions and exclusions. Furthermore, a debate should explore what good work can be accomplished that does not prevent health but creates it (Antonovsky, 1997) and whether this may be considered a science that assumes that people always move along the continuum between good health and illness, thereby taking it into account in its structures (Antonovsky, 1997). The follow-up perspectives raised awareness to the dynamic intertwining of infrastructure, resources, authorization, and empowerment in the university context. In the context of the findings of Butler (2005), we should consider what is (or is not) recognized as being sick/healthy or as an injury. This question is also a political one with individual consequences of participation and vulnerabilities.

Overall, it can be seen that the body of the academic deserves more organizational care. Evidently, these challenges are largely individualized and poorly anchored in existing structures. It is the responsibility of science to address such challenges. The stability of the body should not only be questioned when it is actually absolutely injured (no longer “functions”), as in the case of a broken vertebra, a migraine attack, or depression. Rather, we should assume that we are on a continuum between absolutely injured (ill) and absolutely not injured (healthy), but we are actually always balancing ourselves on the beam of the continuum. To become/be healthy, we also need a framework in which new governmental self-images can be withdrawn. This is because the hegemonic (labor) norm of the “flexible, efficient, fit, and consuming body in post-Fordist conditions” (Graf, 2013) leaves no room for this continuum, and its normative demands generate considerable stress and pressure, which leads to creative processes being stifled by enormous self-pressure and certainly does not promote well-being. Bodies must not only be seen as capital for performance. Their vulnerability must also be recognized.

The study has limitations due to its qualitative approach. A quantitative follow-up questions could measure the prevalence, intensity, and statistical relationships of experiences among scientists with caregiving responsibilities in the context of digitalized work. These might include the number of hours spent using digital tools, the extent to which digitalization facilitates or increases work demands, weekly hours of caregiving, perceived interference of care responsibilities with professional tasks, and self-reported stress or health outcomes. Additional questions could assess the ability to manage flexible working arrangements, maintain boundaries between work and private life, and demographic factors such as gender, career stage, or discipline. Collecting such data allows researchers to identify correlations between digital work practices, caregiving responsibilities, and well-being, compare subgroups, and quantify trends that emerge from qualitative findings, thereby providing a broader and statistically grounded perspective.

Finally, it should be noted once again that, due to the characteristics described at the outset, the field of science can be compared to other professional fields such as industry. However, this is precisely where one of the main problems lies: the neoliberal and increasingly market-oriented organization of scientific activity, which effectively turns scientists into entrepreneurs means that scientists are increasingly subject to the economic constraints of a free market economy. From the perspective of academic freedom, this leads to critical developments which, although promising a high degree of innovation due to ongoing self-marketing, further exacerbate social inequalities through selection pressure, which has a negative impact on scientists with care responsibilities in intersection with other dimensions of inequality (e.g., gender, ability). The comparability of science with sectors such as industry is therefore a development that warrants consideration and should be viewed critically.

## Data Availability

The original contributions presented in the study are included in the article/supplementary material, further inquiries can be directed to the corresponding author.

## References

[ref1] Acker J. (1990). Hierarchies, jobs, bodies: a theory of gendered organizations. Gend. Soc. 4, 139–158. doi: 10.1177/089124390004002002

[ref2] Acker J. (2012). Gendered organizations and intersectionality: problems and possibilities. Equal. Divers. Incl. An. Int. J. 31, 214–224. doi: 10.1108/02610151211209072

[ref4] Anderson B. (2000). Doing the dirty work? The global politics of domestic labour. London: Palgrave Macmillan.

[ref5] Antonovsky A. (1997). Salutogenese. Zur Entmystifizierung der Gesundheit. Tübingen: DGVT-Verlag.

[ref6] Ashforth B. E. Kreiner G. E. Fugate M. (2000). All in a day’s work: boundaries and micro role transitions. Acad. Manag. Rev. 25, 472–491. doi: 10.2307/259305

[ref11] Banday M. U. L. (2025). Enterprising subjects and gendered-ageing: economization of gendered life course and career temporalities among Indian information technology employees. Cult. Organ., 1–20. doi: 10.1080/14759551.2025.2508320, PMID: 40989069

[ref12] Barkhuizen N. Rothmann S. van de Vijver F. J. R. (2014). Burn-out and work engagement of academics in higher education institutions: effects of dispositional optimism. Stress. Health 30, 322–332. doi: 10.1002/smi.2520, PMID: 23949954

[ref13] Barry K. M. Woods M. Warnecke E. Stirling C. Martin A. (2018). Psychological health of doctoral candidates, study-related challenges and perceived performance. High. Educ. Res. Dev. 37, 468–483. doi: 10.1080/07294360.2018.1425979

[ref14] Beckel J. L. O. Fisher G. G. (2022). Telework and worker health and well-being: a review and recommendations for research and practice. Int. J. Environ. Res. Public Health 19:3879. doi: 10.3390/ijerph19073879, PMID: 35409563 PMC8998114

[ref15] Becker-Schmidt R. (1987). “Die doppelte Vergesellschaftung – die doppelte Unterdrückung: Besonderheiten der Frauenforschung in den Sozialwissenschaften” in Unterkirchen, Lilo/Wagner, Ina (Hg.): Die andere Hälfte der Gesellschaft (Wien: Österreichischer Soziologentag), 10–25.

[ref16] Berry C. Valeix S. Niven J. Chapman L. Roberts P. Hazell C. (2020). Hanging in the balance: conceptualising doctoral researcher mental health as a dynamic balance across key tensions characterising the PhD experience. Int. J. Educ. Res. 102:101575. doi: 10.1016/j.ijer.2020.101575

[ref17] Bohnsack R. (2000). “Documentary method and group discussions” in Qualitative analysis and documentary method in international educational research. eds. Bohnsack R. Pfaff N. Weller W. (Opladen: Budrich), 99–124.

[ref18] Bohnsack R. Nentwig-Gesemann I. Nohl A.-M. (2013). Die dokumentarische Methode und ihre Forschungspraxis. Wiesbaden: Springer.

[ref19] Bourdieu P. (1988). Homo academicus. Stanford, CA: Stanford University Press.

[ref20] Bourdieu P. (2002). Masculine domination. Stanford, CA: Stanford University Press.

[ref21] Bröckling U. (2007). Das unternehmerische Selbst. Berlin: Suhrkamp.

[ref22] Brown N. Leigh J. (2018). Ableism in academia: where are the disabled and ill academics? Disabil. Soc. 33, 985–989. doi: 10.1080/09687599.2018.1455627

[ref23] Brückner M. (2010). Entwicklungen der Care-Debatte. Wurzeln und Begrifflichkeiten. In: Apitzsch U./ Schmidbaur M., Care und Migration. Die Ent-Sorgung menschlicher Reproduktionsarbeit entlang von Geschlechter- und Armutsgrenzen (Opladen, Farmington Hills: Verlag Barbara Budrich), 43–58.

[ref24] Buomprisco G. Ricci S. Perri R. De Sio S. (2021). Health and telework: new challenges after COVID-19 pandemic. Eur. J. Environ. Public Health 5:9705. doi: 10.21601/ejeph/9705

[ref25] Burian J. Radtke J. Schulte J. (2022). Stress und Zeitdruck – Situation von wissenschaftlichen Mitarbeitenden seit COVID-19. DGVU Forum, Fachmedium für Prävention, Rehabilitation, Versicherungsrecht, Forschung 2022, 21–27.

[ref26] Butler J. (2001). Psyche der Macht. Das Subjekt der Unterwerfung. Frankfurt/M: Suhrkamp.

[ref27] Butler J. (2005). Gefährdetes Leben. Politische Essays. Frankfurt/M: Suhrkamp.

[ref28] Campbell F. (2009). Contours of ableism. The production of disability and Abledness. Basingstoke: Palgrave Macmillan UK.

[ref29] Carstensen T. (2016). Social Media in der Arbeitswelt. Herausforderungen für Beschäftigte und Mitbestimmung. Bielefeld: Transcript.

[ref30] Castro-Trancon N. Zuazua-Vega M. Osca A. Cifre E. García-Izquierdo A. L. (2024). Effects of teleworking on wellbeing from a gender perspective: a systematic review. Front. Organ. Psychol. 2:1360373. doi: 10.3389/forgp.2024.1360373

[ref31] Chirico F. Zaffina S. Di Prinzio R. R. Giorgi G. Ferrari G. Capitanelli I.. (2021). Working from home in the context of COVID-19: a systematic review of physical and mental health effects of teleworkers. J. Health Soc. Sci. 6, 319–332. doi: 10.19204/2021/wrkn8

[ref32] Connell R. (1987). Gender and power: Society, the person and sexual politics. Stanford, CA: Stanford University Press.

[ref33] Crawford J. (2022). Working from home, telework, and psychological well-being? A systematic review. Sustainability 14:11874. doi: 10.3390/su141911874

[ref34] Currie J. Eveline J. (2011). E-technology and work/life balance for academics with young children. High. Educ. 62, 533–550. doi: 10.1007/s10734-010-9404-9

[ref35] Esteves L. S. Crabtree S. A. Hemingway A. (2020). Impacts of C-19 lockdown on the work-life balance of BU academics: Preliminary results. Available online at: http://eprints.bournemouth.ac.uk/34070/1/Preliminary%20results%20C-19%20impact%20on%20work-life%20balance%20of%20BU%20academics.pdf (Accessed May 31, 2025).

[ref36] Felfe J. Krick A. Hauff S. Renner K.-H. Klebe L. Schübbe K.. (2022). “Working from home: opportunities and risks for working conditions, leadership and health,” in dtec.bw–Beiträge der Helmut-Schmidt-Universität, Universität der Bundeswehr Hamburg – Forschungsaktivitäten im Zentrum für Digitalisierungs- und Technologieforschung der Bundeswehr dtec.bw., eds. Schulz D. Fay A. Schulz M.. (Hamburg: Helmut-Schmidt-Universität, Universität der Bundeswehr), 335–341.

[ref37] Fonner K. L. Roloff M. E. (2010). Why teleworkers are more satisfied with their jobs than are office-based workers: when less contact is beneficial. J. Appl. Commun. Res. 38, 336–361. doi: 10.1080/00909882.2010.513998

[ref38] Foucault M. (1977). Überwachen und Strafen. Die Geburt des Gefängnisses. Frankfurt/M: Suhrkamp.

[ref39] Furuya Y. Nakazawa S. Fukai K. Tatemichi M. (2022). Health impacts with telework on workers: a scoping review before the COVID-19 pandemic. Front. Public Health 10:981270. doi: 10.3389/fpubh.2022.981270, PMID: 36388332 PMC9660232

[ref40] Getto B. Zellweger F. (2021). “Entwicklung von Studium und Lehre in der Pandemie: Strategische Diskurse im Kontext der Digitalisierung” in Bildung in der digitalen Transformation. eds. Wollersheim H.-W. Karapanos M. Pengel N. (Münster; New York: Waxmann), 173–178.

[ref41] Gill R. Scharff C. (2011). New femininities: Postfeminism, neoliberalism and subjectivity. Basingstoke: Palgrave.

[ref42] Glaser B. Strauss A. (1967). The discovery of grounded theory: Strategies for qualitative research. Chicago, IL: Aldine Publishing Company.

[ref43] Graf S. (2013). Leistungsfähig, attraktiv, erfolgreich, jung und gesund: der fitte Körper in post-fordistischen Verhältnissen. Body Polit. 1, 139–157. doi: 10.12685/bp.v1i1.1434

[ref44] Gutierrez-Rodriguez E. (2014). Domestic work–affective labor: on feminization and the coloniality of labor. Women’s Stud. Int. Forum 46, 45–53. doi: 10.1016/j.wsif.2014.03.005

[ref7] Haag H. Gamper M. (2022). Wenn‘s nirgendwo so richtig stimmt“. Einblicke in qualitative Forschung zu Hochschulkarrieren und Elternschaft unter Corona-Bedingungen. Femina Politica –zeitschrift für feministische Politikwissenschaft, 31, 132–136.

[ref8] Haag H. Gamper M. (2025). New Fathers – New Care – New Work. Leaving “Homo Academicus”? Schweizer Zeitschrift für Soziologie, 51. doi: 10.26034/cm.sjs.2025.6196

[ref10] Haag H. Schmitt S. Reuter J. (2024). Auf dem Weg vom ideal academic worker zum caring academic worker. Potentiale von Care für eine diverse(re) Hochschule. Zeitschrift für Diversitätsforschung und -management, Schwerpunktheft: Care & Diversity aus intersektionaler Perspektive. 71–76. doi: 10.3224/zdfm.v9i1.08

[ref45] Heiden M. Widar L. Wiitavaara B. Boman E. (2021). Telework in academia: associations with health and well-being among staff. High. Educ. 81, 707–722. doi: 10.1007/s10734-020-00569-4

[ref46] Hengelaar A. H. Wittenberg Y. Kwekkeboom R. Van Hartingsveldt M. Verdonk P. (2021). Intersectionality in informal care research: a scoping review. Scand. J. Public Health 51, 106–124. doi: 10.1177/14034948211027816, PMID: 34232094 PMC9903248

[ref47] Hirsch-Kreinsen H. Ittermann P. Niehaus J. (2015). Digitalisierung industrieller Arbeit. Die Vision Industrie 4.0 und ihre sozialen Herausforderungen. Baden-Baden: Nomos Verlag.

[ref48] Jurczyk K. (2014). “Entgrenzte Arbeit und Care in privaten Lebensformen” in Für sich und andere sorgen. Krise und Zukunft von Care in der modernen Gesellschaft. eds. Aulenbacher B. Dammayr M. (Weinheim: Beltz Juventa), 171–182.

[ref49] Kaihlanen A. M. Laukka E. Nadav J. Närvänen J. Saukkonen P. Koivisto J.. (2023). The effects of digitalisation on health and social care work: a qualitative descriptive study of the perceptions of professionals and managers. BMC Health Serv. Res. 23:714. doi: 10.1186/s12913-023-09730-y, PMID: 37386423 PMC10311778

[ref50] Kirschenbauer A. (2015). “Neuformierung von Arbeit und Leben durch Informatisierung? Projektergebnisse – empirische Auswertung” in Geschlechterarrangements in Bewegung. eds. Wischermann U. Kirschenbauer A. (Bielefeld: transcript), 25–117.

[ref51] Kitzinger J. (1994). The methodology of focus groups: the importance of interaction between research participants. Sociol. Health Illn. 16, 103–121. doi: 10.1111/1467-9566.ep11347023

[ref52] Klinger C. (2014). Selbstsorge oder Selbsttechnologie? Das Subjekt zwischen liberaler Tradition und Neoliberalismus. In: Aulenbacher B./ Dammayr M., Für sich und andere sorgen. Krise und Zukunft von Care in der modernen Gesellschaft (Weinheim, Basel: Beltz Juventa), 31–40.

[ref53] Knapp G.-A. (1990). “Zur widersprüchlichen Vergesellschaftung von Frauen” in Die doppelte Sozialisation Erwachsener. Zum Verhältnis von beruflichem und privatem Lebensstrang. ed. Hoff E.-H. (München: DJI Verlag, S), 17–52.

[ref54] Kniffin K. M. Narayanan J. Anseel F. Antonakis J. Ashford S. P. Bakker A. B.. (2021). COVID-19 and the workplace: implications, issues, and insights for future research and action. Am. Psychol. 76, 63–77. doi: 10.1037/amp0000716, PMID: 32772537

[ref55] Krick A. Arnold M. Felfe J. (2024). Self-care when working from home: easier but also more important. Front. Organ. Psychol. 2:1333689. doi: 10.3389/forgp.2024.1333689

[ref57] Laß I. Rüger H. (2024). Homeoffice und das Wohlbefinden von Eltern während der Coronapandemie. Available online at: https://www.bpb.de/kurz-knapp/zahlen-und-fakten/sozialbericht-2024/553175/homeoffice-und-das-wohlbefinden-von-eltern-waehrend-der-coronapandemie/ (Accessed May 31, 2025).

[ref58] Leal Filho W. Wall T. Rayman-Bacchus L. Mifsud M. Pritchard D. J. Lovren V. O.. (2021). Impacts of COVID-19 and social isolation on academic staff and students at universities: a cross-sectional study. BMC Public Health 21:1213. doi: 10.1186/s12889-021-11040-z, PMID: 34167494 PMC8223197

[ref59] Maihofer A. (1995). Geschlecht als Existenzweise. Sulzbach/Taunus: Ulrike Helmer Verlag.

[ref60] Maihofer A. (2021). “Wandel und Persistenz hegemonialer Männlichkeit – aktuelle Entwicklungen” in AG Transformationen Männlichkeiten. ed. Schweiz Z. M. (Zürich: Genf: Seismo), 31–54.

[ref61] Mayring P. (2007). Qualitative Inhaltsanalyse. Grundlagen und Techniken. Weinheim: Beltz.

[ref62] McRobbie A. (2009). The aftermath of feminism: Gender, culture and social change. London: Sage.

[ref63] Mirchandani K. (1999). Legitimizing work: telework and the gendered reification of the work-nonwork dichotomy. Can. Rev. Sociol. 36, 87–107. doi: 10.1111/j.1755-618X.1999.tb01271.x

[ref64] Morgan D. L. (1997). Focus groups as qualitative research. New York: Sage.

[ref65] Nicholls H. Nicholls M. Tekin S. Lamb D. Billings J. (2022). The impact of working in academia on researchers’ mental health and well-being: a systematic review and qualitative metasynthesis. PLoS One 17:5. doi: 10.1371/journal.pone.0268890, PMID: 35613147 PMC9132292

[ref66] Nickel H. M. (2015). ““Vermarktlichung” und “Subjektivierung”: eine widersprüchliche und spannungsreiche Rahmung für Geschlechterverhältnisse” in Geschlechterverhältnisse im Post-Wohlfahrtsstaat. eds. Nadai E. Nollert E. M. (Weinheim/Basel: Beltz Juventa), 28–48.

[ref67] Niebuhr F. Borle P. Börner-Zobel F. Voelter-Mahlknecht S. (2022). Healthy and happy working from home? Effects of working from home on employee health and job satisfaction. Int. J. Environ. Res. Public Health 19:1122. doi: 10.3390/ijerph19031122, PMID: 35162145 PMC8834350

[ref68] Paulitz T. Wagner L. (2020). Professorinnen – jenseits der Gläsernen Decke? Eine qualitative empirische Studie zu geschlechtshierarchisierenden Praxen der Alltagskultur an Hochschulen. GENDER – Z. Geschlecht, Kultur Gesellsch. 12:133148:2. doi: 10.3224/gender.v12i2.09

[ref69] Percival J. Vogel E. Muirhead B. (2011). Telecommuting in higher education: faculty perceptions of strategic implications for traditional postsecondary institutions. Int. J. Manag. Educ. 5, 271–284. doi: 10.1504/IJMIE.2011.039489

[ref70] Plessner H. (1981). “Gesammelte Schriften” in Band IV: Die Stufen des Organischen und der Mensch (Einleitung in die philosophische Anthropologie. Frankfurt/M: Suhrkamp).

[ref71] Pongratz H. J. Voß G. G. (2004). Arbeitskraftunternehmer. Erwerbsorientierungen in entgrenzten Arbeitsformen. 2nd Edn. Berlin: edition sigma.

[ref72] Radtke J. S. Burian J. (2021). “Der Arbeitsplatz Hochschule in Zeiten von Corona: Arbeitsbedingungen und Gesundheit in Wissenschaft und Verwaltung” in Fehlzeiten-Report 2021. eds. Badura B. Ducki A. Schröder H. Meyer M. (Cham: Springer), 123–148.

[ref73] Rosak-Szyrocka J. Żywiołek J. Zaborski A. Chowdhury S. Hu Y.-C. (2022). Digitalization of higher education around the globe during COVID-19. IEEE Access 10, 59782–59791. doi: 10.1109/ACCESS.2022.3178711

[ref74] Scharff C. (2016). “Gender and neoliberalism: young women as ideal neoliberal subjects” in Handbook of neoliberalism (eds.) Springer S Birch K MacLeavy J (London: Routledge), 217–226.

[ref75] Schnabel P.-E. (2006). “Zur Gesundheit des wissenschaftlichen Personals an Hochschulen – vermeidbare Belastungen erkennen und Potenziale fördern” in Wege zur gesunden Hochschule. Ein Leitfaden für die Praxis. eds. Faller G. Schnabel P.-E. (Berlin: edition sigma), 141–159.

[ref76] Schnabl C. (2005). Gerecht sorgen. Grundlagen einer sozialethischen Theorie der Fürsorge. Freiburg i.Br., Wien: Herder.

[ref77] Sevic A. Lungu D. A. Brønnick K. K. (2025). In the shadows of digitalisation: digital stressors as predictors of emotional exhaustion in Norwegian academia. Behav. Inform. Technol. 44, 4372–4385. doi: 10.1080/0144929X.2025.2472942

[ref78] Shalaby M. Allam N. Buttorff G. J. (2021). Leveling the field: gender inequity in academia during COVID-19. PS Polit. Sci. Polit. 54, 661–667. doi: 10.1017/S1049096521000615

[ref79] Stadelbacher S. (2010). “Die klassische Soziologie und der Körper: handlungstheoretische Zugänge und ihr Verhältnis zur Körperlichkeit der Akteure” in Die Körperlichkeit sozialen Handelns. Soziale Ordnung jenseits von Normen und Institutionen. eds. Böhle F. Weihrich M. (Bielefeld: transcript), 35–58.

[ref80] ten Brummelhuis L. L. ter Hoeven C. L. Toniolo-Barrios M. (2021). Staying in the loop: is constant connectivity to work good or bad for work performance? J. Vocat. Behav. 128:103589. doi: 10.1016/j.jvb.2021.103589, PMID: 41080625

[ref81] Vacchiano M. Fernandez G. Schmutz R. (2024). What’s going on with teleworking? A scoping review of its effects on well-being. PLoS One 19:e0305567. doi: 10.1371/journal.pone.0305567, PMID: 39159254 PMC11332997

[ref82] van Dick R. Baethge A. Junker N. M. (2024). Editorial: implications of remote work on employee well-being and health. Front. Organ. Psychol. 2:1498944. doi: 10.3389/forgp.2024.1498944

[ref83] Väth M. (2016). “Arbeit – die schönste Nebensache der Welt” in Wie New Work unsere Arbeitswelt revolutioniert (Offenbach: GABAL Verlag).

[ref84] Wagner H. (2013). “Chancen für eine emanzipatorische Arbeits- und Geschlechterpolitik? Aus gewerkschaftlicher Perspektive” in Krise, Kritik, Allianzen. Arbeits- und geschlechtersoziologische Perspektiven. eds. Nickel H. M. Heilmann A. (Weinheim: Beltz Juventa), 202–218.

[ref85] Warmuth A.-D. Glockentöger I. (2018). “Effects of digitalized and flexible workplaces on parenthood: new concepts in gender relations or a return to traditional gender roles?” in The impact of digitalization in the workplace. An educational view. ed. Harteis C. (Cham: Springer), 71–86.

[ref86] Watermeyer R. Crick T. Knight C. Goodall J. (2021). COVID-19 and digital disruption in UK universities: afflictions and affordances of emergency online migration. High. Educ. 81, 623–641. doi: 10.1007/s10734-020-00561-y, PMID: 32836334 PMC7269686

[ref87] Wegrzyn E. Altenstädter L. Alberg I. Ötztas S. Yilmaz B. (2021). Sorgearbeit und Qualifizierung in der Wissenschaft in Zeiten von Corona – Einblicke in qualitative Forschung zu Juniorprofessuren. Femina Polit. 2, 193–197. doi: 10.3224/feminapolitica.v30i2.20

[ref88] Wichterich C. (2023). “Global political economy of care and gender-crisis, extractivism and contestation” in Handbook of research on the global political economy of work. (eds.) Atzeni M Azzellini D Mezzadri A Moore P Apitzsch U. (Edward Elgar Publishing), 401–411.

[ref89] Widar L. Heiden M. Boman E. Wiitavaara B. (2022). How is telework experienced in academia? Sustainability 14:5745. doi: 10.3390/su14105745

[ref90] Yildirim T. M. Eslen-Ziya H. (2021). The differential impact of COVID-19 on the work conditions of women and men academics during the lockdown. Gend. Work. Organ. 28, 243–249. doi: 10.1111/gwao.12529, PMID: 32904915 PMC7461380

[ref91] Zimmermann A. (2022). Von hegemonialen Erfolgsgeschichten zu Männlichkeiten in Transformation. GENDER – Z. Geschlecht, Kultur Gesellsch 14, 57–72. doi: 10.3224/gender.v14i2.05

[ref92] Zimmermann A. Weibel F. (2020). Von “diversity management” zu “diversity und inclusion”? ZDfm 5, 153–166. doi: 10.3224/zdfm.v5i2.02

